# Body mass index in an Australian population with chronic kidney disease

**DOI:** 10.1186/s12882-018-1006-2

**Published:** 2018-08-20

**Authors:** Samuel Chan, Anne Cameron, Zaimin Wang, Sree K. Venuthurupalli, Ken S. Tan, Helen G. Healy, Wendy E. Hoy

**Affiliations:** 1 Kidney Health Service, Royal Brisbane and Women’s Hospital, Metro North Hospital and Health Service, Brisbane, QLD Australia; 2 CKD.QLD and the NHMRC CKD.CRE, The University of Queensland, Brisbane, QLD Australia; 3 Faculty of Medicine, The University of Queensland, Brisbane, QLD Australia; 4 Renal Services, Toowoomba Hospital, Toowoomba, QLD Australia; 5 Department of Nephrology, Logan Hospital, Logan, QLD Australia

**Keywords:** Associations, Body mass index, Chronic kidney disease, Clinical, Demographics

## Abstract

**Background:**

Obesity emerged as the leading global health concern in 2017. Although higher body mass index (BMI) is a health risk in the general population, its implications for chronic kidney disease (CKD) are not entirely clear. Our aim was to compare BMI in an Australian CKD population with BMI in a sample of the general Australian population, and, in the same group of CKD patients, to describe associations of higher BMI categories with demographic and clinical features.

**Methods:**

A cross-sectional study of BMI in CKD patients was conducted from three major sites who were enrolled in the CKD.QLD registry between May 2011 and July 2015. BMI was categorized according to the World Health Organisation (WHO) guidelines. The prevalence of obesity was compared with a sample of the general Australian population from the most recent National Health Survey (NHS). Associations of BMI with demographic and clinical characteristics of the CKD patients were also analysed.

**Results:**

There were 3382 CKD patients in this study (median age 68, IQR 56–76 years); 50.5% had BMI ≥30, the WHO threshold for obesity, in contrast with 28.4% having BMI ≥30 in the NHS cohort. Higher BMI categories were correlated with age < 70 years, male gender, and lower socioeconomic status. After adjustment for age and gender, characteristics which significantly correlated with higher BMI category included hypertension, dyslipidemia, diabetes, diabetic nephropathy, coronary heart disease, other cardiovascular diseases, gout, obstructive sleep apnoea, depression and chronic lung disease.

**Conclusions:**

Patients with CKD in public renal specialty practices in Queensland have strikingly higher rates of obesity than the general Australian population. Within the CKD population, low socio-economic position strongly predisposes to higher BMI categories. Higher BMI categories also strongly correlated with important co-morbidities that contribute to burden of illness. These data flag major opportunities for primary prevention of CKD and for reductions in morbidity in people who already have CKD, which should be considered in public health policy in relation to obesity.

## Background

Addressing obesity was the theme of World Kidney Day in 2017 [1]. Obesity is associated with marked reduction in life expectancy, and an increased risk of morbidity and mortality from type II diabetes mellitus, respiratory disease, musculoskeletal conditions such as gout and osteoarthritis, as well as cancer and infertility [2, 3]. Body mass index (BMI) has also been proposed to drive the development and/or progression of chronic kidney disease (CKD) [4, 5]. CKD is defined as glomerular filtration rate (GFR) less than 60 mL/min/1.73 m2 for three or more months with or without kidney damage, or kidney damage for three or more months as defined by structural or functional abnormalities of the kidney, with or without decreased GFR, manifested by either pathological abnormalities, or markers of kidney damage including abnormalities in the blood or urine, or abnormalities in imaging tests.

The relationship between BMI and CKD has been contested. Earlier studies have suggested that a higher BMI was associated with an increased risk of developing CKD [6–9], whilst other investigations found no association between BMI and CKD [10–14]. A 2017 meta-analysis showed that a higher BMI predicted the onset of albuminuria in all stages of CKD, but the effect was significant only in obese individuals [15]. There is no conclusive evidence that the impact of obesity in the CKD population is the same as in the general population without CKD, where most of the health association research has been performed. Also unknown are the contributions of patient demographics, clinical phenotype or a combination of both, to the obesity signal in CKD.

In this study, we described BMI and BMI categories in patients with CKD in public renal specialty practices in Queensland. In addition, we compared this with an aged-matched contemporaneous Australian population. Furthermore, we evaluated the associations of BMI categories in the CKD population with patient demographic and clinical characteristics.

## Methods

This study is a cross-sectional evaluation of participants from the CKD.QLD Registry. CKD.QLD has been described elsewhere [16, 17]. In brief, it comprises a multidisciplinary research and practice collaborative network embracing Queensland Health nephrology services, as well as academics from the Centre for Chronic Disease, The University of Queensland and the Queensland University of Technology. There are currently 8641 persons recruited to the CKD.QLD cohort as of May 2018. All patients in the cohort had been referred to a specialist nephrologist in the Queensland public health system and had a diagnosis of CKD ascribed by a nephrologist in that system. Additionally, written consent to participate in the CKD.QLD Registry was obtained from the participants. Only sites that had recruited more than three quarters of their CKD population into the CKD.QLD Registry were included in this study on BMI and obesity.

### Participants

Patients from three sites were included in this study – Logan Hospital (Logan), Royal Brisbane and Women’s Hospital (RBWH), and Toowoomba Base Hospital (Toowoomba). The total number of CKD patients was 3382, who had all been enrolled in the registry between May 2011 and June 2015.

### Data collection

Baseline data on each individual, captured at time of enrolment into CKD.QLD, were extracted from the CKD.QLD Registry. Demographic characteristics included age, gender, indigenous ethnicity, socio-economic position (using the Index of Relative Socio-economic Disadvantage (IRSD) [18], and BMI. BMI (kg/m^2^) was stratified into the five World Health Organisation categories: normal BMI was defined as BMI < 25; overweight was defined as BMI 25 to < 30; Class I obesity was defined as BMI 30 to < 35, Class II obesity was defined as BMI 35 to < 40, and Class III obesity was defined as BMI ≥40 [19]. IRSD has been developed as part of the Socio-Economic Indices for Areas by the Australian Bureau of Statistics. This index was allocated by postcode and based on a number of characteristics, including household income, employment and education level. Socio-economic groups were grouped in quintiles, with Quintile 1 representing the 20% of the general Australian population within the lowest socio-economic characteristics and Quintile 5 having the highest socio-economic characteristics. Kidney function was recorded as the estimated glomerular filtration rate (eGFR) at time of consent and transformed to stage of CKD, according to the National Kidney Foundation classification system [20]. CKD aetiology was recorded using the coding of the Australian New Zealand Dialysis and Transplant (ANZDATA) registry [21]. Co-morbidities at the time of consent to the registry, as documented by health care providers, included hypertension, diabetes mellitus, coronary artery disease, other cardiovascular diseases, gout, obstructive sleep apnoea, chronic lung disease and depression.

The 2014–15 National Health Survey (NHS) is the most recent in a series of Australia-wide health surveys conducted by the Australian Bureau of Statistics [22]. The survey was distributed across urban, rural and remote areas of Australia from July 2014 to June 2015 and included approximately 19,000 people in nearly 15,000 private dwellings. The age range of the participants was between 18 and 90 years.

BMI was calculated using the reported height and weight in the NHS study. For the CKD.QLD patients, the BMI was recorded from the clinical records as close as possible to the time of patient recruitment to the Registry.

### Statistical analyses

Results were expressed as frequencies (percentages) for categorical data, mean (standard deviation) for continuous normally distributed data or median (interquartile range) for continuous non-normally distributed data. The prevalence of obesity was compared with the sample of the Australian general population from the 2014–15 NHS. Age was dichotomised into age < 70 years and ≥ 70 years. Then, among the pooled data of the CKD.QLD patients, the associations of BMI category with various factors, including the ten most common complications and co-morbidities, were evaluated, via a multivariable model using logistic regression, adjusting for age and gender. All analyses were undertaken using Stata 14.1 (Stata Corp. Stata Statistical Software: Release 14.1, College Station. TX: StatCorp LP, 2016). Statistical significance was defined as a *p*-value < 0.05 (two-tail).

This study was approved by the Human Research Ethics Committee, Royal Brisbane and Women’s Hospital, Queensland Health – HREC/15/QRBW/294 – and the Medical Research Ethics Committee, University of Queensland – number 2011000029, with subsequent protocol amendments and study extensions as required.

## Results

**Fig. 1:**
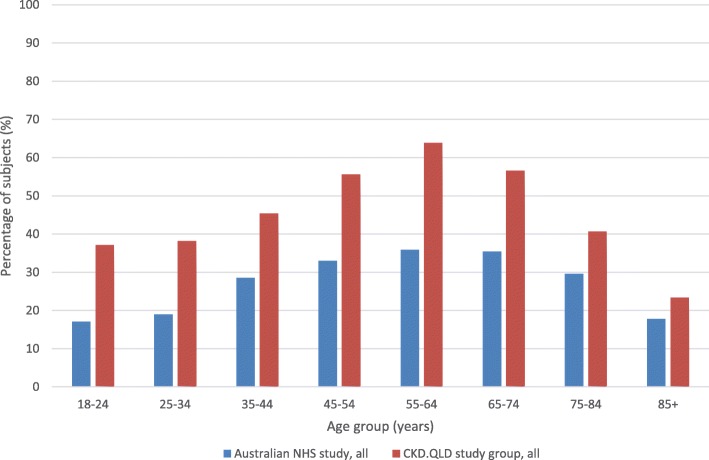
Proportions of subjects with BMI > 30 kg/m2 by age group, Australian National Health Survey (2014) versus CKD.QLD patients

Table 1 summarises the demographic and clinical characteristics of the CKD.QLD Registry patient cohort at time of consent. Among the 3382 patients in this study, the median BMI was 30.0 kg/m^2^ (IQR 26–35.1 kg/m^2^) with 50.5% of the cohort having BMI ≥30, the threshold of WHO classification for obesity. This contrasts with a median BMI of 25.9 kg/m^2^ in participants in the 2014–15 NHS, in which 28.4% of the participants had BMI ≥30. Figure 1 compares, by age group, the proportions of subjects with obesity in the NHS with those of the CKD patients in aggregate. The CKD population had higher proportions of obesity than the NHS population at every age group, and, within the CKD population, those with diabetes (45.5% of the whole CKD cohort) had higher proportions of obesity than those without diabetes. Compared with the rate of 28.4% in the NHS population overall, the proportion of obesity was 38% in the CKD population without diabetes (OR 2.40, CI 1.92–2.57), 50.5% in the CKD population overall (OR 2.72, CI 2.31–2.93) and 65% in the CKD population with diabetes (OR = 4.42, CI 4.02–4.85).

**Table 1:** CKD.QLD Registry patient characteristics at time of consent

Characteristics	Total number (*n* = 3382)
Age ≥ 70 years	1515 (44.8%)
Male gender	1783 (52.7%)
Indigenous ethnicity	120 (4%)
Index of relative socio-economic disadvantage	
Lowest	791 (23%)
Highest	472 (14%)
Body mass index (BMI)	
Normal BMI	670 (19.8%)
Overweight BMI	1001 (29.6%)
Obese BMI	840 (24.8%)
Grossly obese BMI	475 (14.0%)
Morbidly obese BMI	393 (11.7%)
Stage of chronic kidney disease	
Stage 1	241 (7.1%)
Stage 2	402 (11.9%)
Stage 3A	610 (18.0%)
Stage 3B	1076 (31.8%)
Stage 4	826 (24.4%)
Stage 5	223 (6.7%)
Aetiology of chronic kidney disease	
Renovascular	1027 (31.2%)
Diabetic nephropathy	801 (24.3%)
Other diagnoses	656 (19.9%)
Glomerulonephritis	435 (13.2%)
Genetic renal disease	200 (6.1%)
Uncertain diagnoses	176 (5.3%)
Co-morbidities	
Hypertension	2728 (80.7%)
Dyslipidemia	1741 (53.0%)
Diabetes mellitus	1538 (45.5%)
Coronary artery disease	846 (25.0%)
Other cardiovascular disease	1350 (39.9%)
Gout	638 (19.4%)
Obstructive sleep apnoea	392 (11.9%)
Chronic lung disease	763 (22.6%)
Depression	440 (13.4%)

**Fig. 2:**
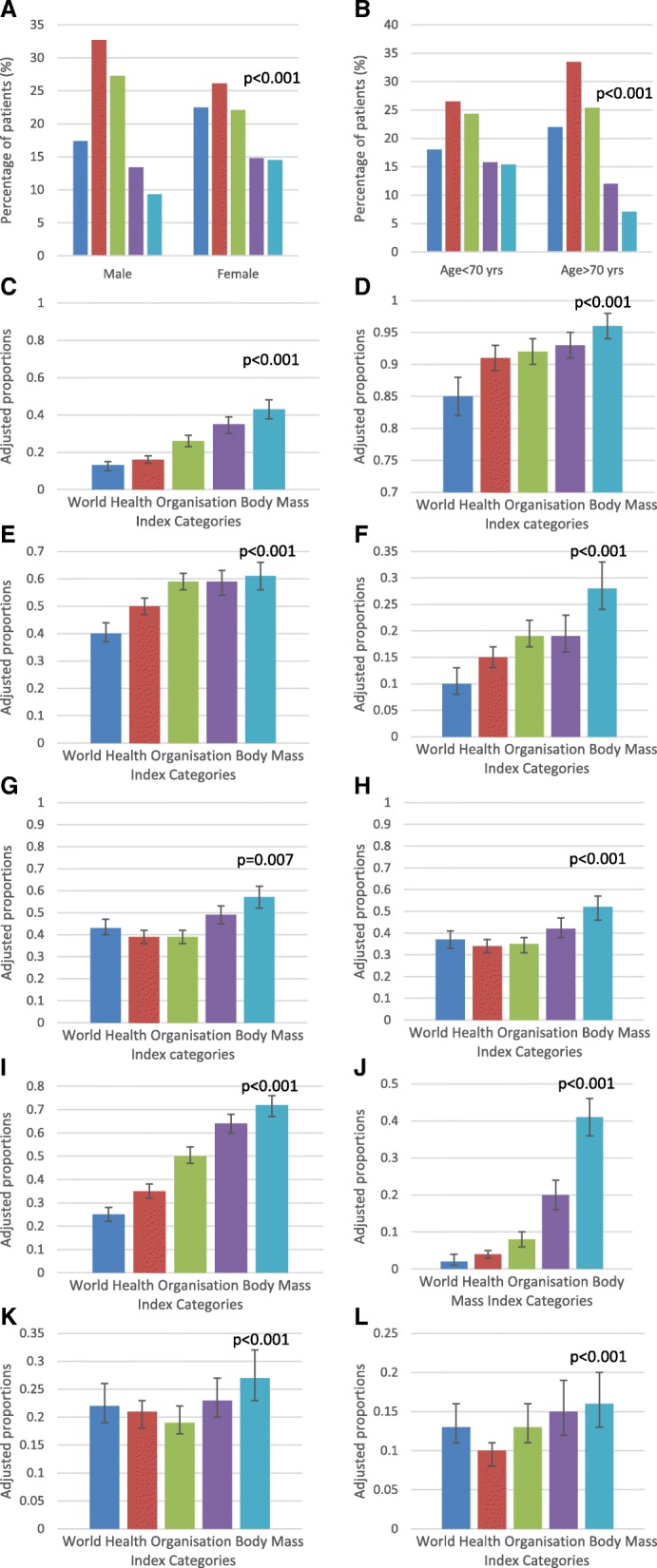
Adjusted proportion of CKD.QLD patients by World Health Organisation Body Mass Index categories by **a** gender; **b** age group; **c** diabetic nephropathy; **d** hypertension; **e** dyslipidaemia; **f** gout; **g** coronary artery disease **h** other cardiovascular diseases; **i** diabetes mellitus; **j** obstructive sleep apnoea; **k** chronic lung disease; and **l** depression

When subjects at all sites were pooled, several characteristics were notably associated with BMI categories. BMI category elevations were higher among males than females (*p* < 0.001) (Fig. 2a), among younger than older CKD patients (< 70 years vs ≥ 70 years old) (*p* < 0.001) (Fig. 2b), and among people in the lowest versus the highest socioeconomic position quintile (*p* < 0.001). Subjects with Stage 5 CKD were less likely to be morbidly obese compared with those with earlier stages of CKD (*p* = 0.018). Proportion of patients with diabetic nephropathy were strongly correlated with higher BMI categories (*p* < 0.001) (Fig. 2c), whereas other common categories of renal disease showed absent or less marked correlations with BMI categories.

Figure 2d to l shows the estimated probabilities of certain co-morbidities and complications predicted by progressively higher categories of BMI, after adjustment for age, sex and site. High rates of hypertension (*p* < 0.001) and dyslipidaemia (*p* < 0.001) were further elevated at higher BMI categories. Appreciable rates of gout (*p* < 0.001), coronary artery disease (*p* = 0.007) and other forms of cardiovascular disease (*p* < 0.001) were moderately, but significantly, correlated with higher BMI categories. Diabetes (*p* < 0.001) as a co-morbidity and obstructive sleep apnoea (*p* < 0.001) were minimised at normal BMI categories and strikingly correlated with progressively higher BMI categories. Finally, the prevalence of chronic lung disease (*p* < 0.001) and depression (*p* < 0.001) were highest in persons in the highest BMI category. We could not define significant associations of higher BMI categories with cancer, cognitive impairment or gastrointestinal complaints in these CKD patients.

## Discussion

To the best of our knowledge, this is the first description of BMI in a population of CKD patients in nephrology specialty practices in Australia. The CKD patients has strikingly higher proportions of obesity than age-matched Australians in a recent National Health Survey. Among the CKD patients, younger age (< 70 years), male gender and lower socioeconomic status were associated with higher levels of obesity. Furthermore, higher levels of obesity were associated with several serious co-morbidities and complications. Higher BMI categories predicted higher levels of hypertension, dyslipidaemia, diabetes, diabetic nephropathy, gout and obstructive sleep apnoea, coronary artery disease and cardiovascular complications. Finally, morbid obesity predicted higher rates of depression and chronic lung disease. Many of these findings are consistent with studies undertaken in CKD population in Norway [6], Japan [7], Malaysia [8] and Sweden [9].

The finding that a greater proportion of participants in stage 5 CKD had a lower BMI may relate to the clinical observation of low protein intake, with hypoalbuminaemia, secondary to anorexia and chronic malnutrition common at this stage of disease, such as occurs in patients on renal replacement therapy [23]. In addition to survival bias in this study, relative nutritional inadequacy may be the underlying mechanism in those aged ≥70 years.

The main strength of this study, being the very large sample size, needs to be balanced against its limitations. First, it is an observational study, examining the cross-sectional relationship between BMI and CKD. Second, participants in this study were those referred to specialist nephrology public system practices, so that findings are not necessarily representative of the general population, or the broader CKD population. Third, residual confounding, potential misclassification and potential selection bias are key limitations of a cross sectional study.

## Conclusion

This study has demonstrated that people who are obese are over-represented in the adult CKD population. Higher BMI categories have been shown to strongly correlate with important co-morbidities that contribute to burden of illness. These data flag major opportunities for primary prevention of CKD and for reductions in morbidity in people who already have CKD, which should be considered in public health policy in relation to obesity. Future studies are required to examine the effect of BMI on time sensitive outcomes such as mortality and progression to renal replacement therapy.
